# An efficient method for the construction of artificial, concatemeric DNA, RNA and proteins with genetically programmed functions, using a novel, vector-enzymatic DNA fragment amplification-expression technology

**DOI:** 10.1016/j.mex.2020.101070

**Published:** 2020-09-21

**Authors:** Piotr M. Skowron, Natalia Krawczun, Joanna Żebrowska, Daria Krefft, Olga Żołnierkiewicz, Marta Bielawa, Joanna Jeżewska-Frąckowiak, Łukasz Janus, Małgorzata Witkowska, Małgorzata Palczewska, Agnieszka Zylicz-Stachula

**Affiliations:** a Department of Molecular Biotechnology, Faculty of Chemistry, University of Gdansk, Gdansk 80-308, Poland; b BioVentures Institute Ltd., Poznan 60-141, Poland

**Keywords:** Artificial proteins, Concatemers, Tandem DNA multimers, Peptide-based biomaterials, DNA fragment amplification, Gene expression, Protein purification

## Abstract

De novo designed bioactive molecules, such as DNA, RNA and peptides, are utilized in increasingly diverse scientific, industrial and biomedical applications. Concatemerization of designed DNA, RNA and peptides may improve their stability, bioactivity and allow for gradual release of the bioactive molecule at the intended destination. In this context, we developed a new method enabling the formation of DNA concatemers for the production of artificial, repetitive genes, encoding concatemeric RNAs and proteins of any nucleotide and amino-acid sequence. The technology recruits the Type IIS SapI restriction endonuclease (REase) for assembling DNA fragments in an ordered head-to-tail-orientation. Alternatively, other commercially available SapI isoschizomers can be used: LguI and thermostable BspQI. Four series of DNA vectors dedicated to the expression of newly formed, concatemeric open reading frames (ORFs), were designed and constructed to meet the technology needs.

• Vector-enzymatic DNA fragment amplification technology.

• Construction of DNA concatemers many times longer than those available with the use of current de novo gene synthesis methods.

• Biosynthesis of protein tandem repeats with programmable function never seen in nature.

## Graphical abstract

**Figure:**
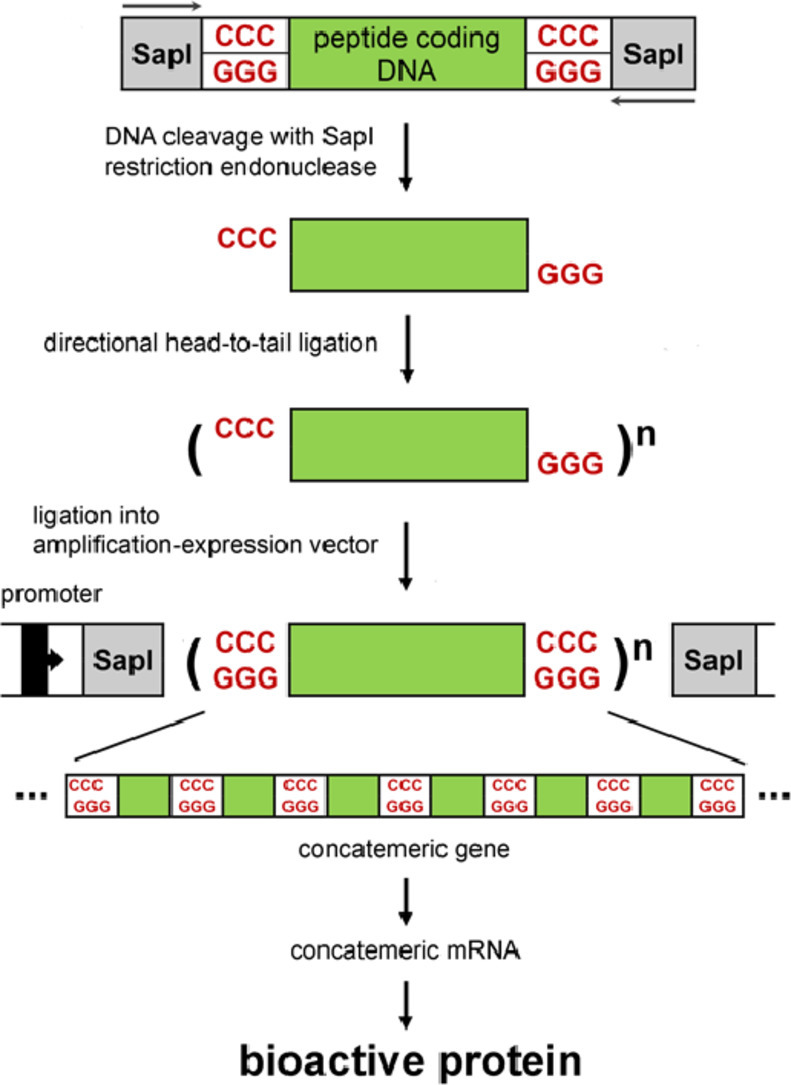

Specifications table

**Table:** 

Subject Area:	Materials Science
More specific subject area	Generation of repetitive genes, encoding concatemeric RNAs and proteins of any nucleotide and amino-acid sequence, which can be used as biomaterials for scientific, industrial and biomedical applications
Method name	Novel, vector-enzymatic DNA fragment amplification-expression technology
Name and reference of original method	Kim, S. and Szybalski, W. (1988). Amplification of cloned DNA as tandem multimers using BspMI-generated asymmetric cohesive ends. Gene, 71(1), 1-8.
Resource availability	P.M. Skowron, N. Krawczun, J. Zebrowska, D. Krefft, O. Zolnierkiewicz, M. Bielawa, J. Jezewska-Frackowiak, L. Janus, M. Witkowska, M. Palczewska, A. Schumacher, A. Wardowska, M. Deptula, A. Czupryn, P. Mucha, A. Piotrowski, P. Sachadyn, S. Rodziewicz-Motowidlo, A. Zylicz-Stachula, A vector-enzymatic DNA fragment amplification-expression technology for construction of artificial, concatemeric DNA, RNA and proteins for novel biomaterials, biomedical and industrial applications, Mater. Sci. Eng. C 108 (2020), 110426. 10.1016/j.msec.2019.110426. P.M Skowron, N. Krawczun, J. Zebrowska; D. Krefft, O. Zolnierkiewicz, M. Bielawa, J. Jezewska-Frackowiak, L. Janus, M. Witkowska, M. Palczewska, A. Schumacher, A. Wardowska, M. Deptula, A. Czupryn, P. Mucha, A. Piotrowski, P. Sachadyn, S. Rodziewicz-Motowidlo, M. Pikula, A. Zylicz-Stachula, Data regarding a new, vector-enzymatic DNA fragment amplification-expression technology for the construction of artificial, concatemeric DNA, RNA and proteins, as well as biological effects of selected polypeptides obtained using this method. Data Brief. 28 (2020), 105069. doi:10.1016/j.dib.2019.105069.

## Introduction

**Fig. 1:**
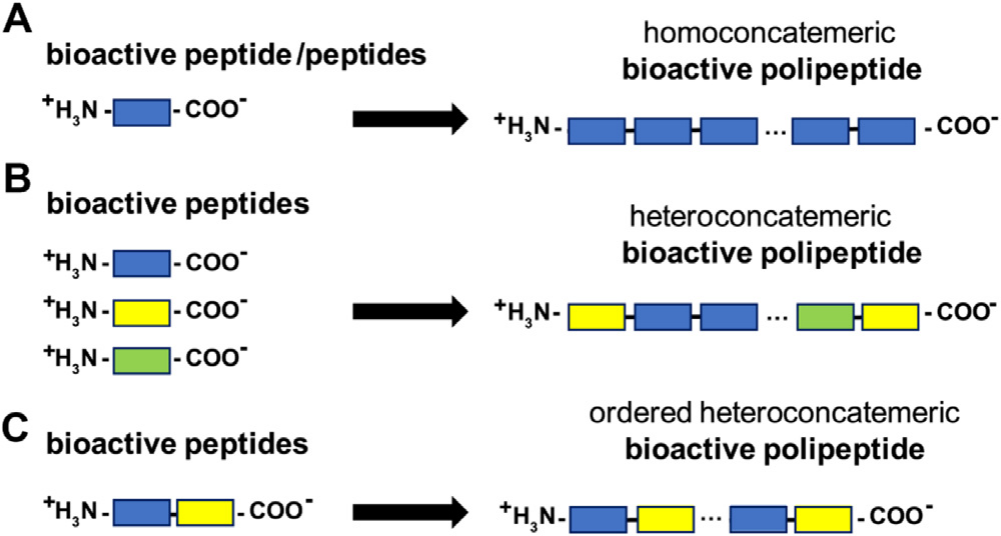
Scheme showing possible products of the developed DNA fragment amplification-expression technology.

Various biomaterials, including those based on peptides and polypeptides are being increasingly developed and used in medicine, as biosensors or in biotechnology and material engineering, among others [1], [2], [3]. Some of these applications rely on specific peptide/polypeptide ligands and can be enhanced by the use of a local concentration increase, e.g. on the molecular level, by generating joined ligands and/or DNA or RNA that encodes them. Example benefits include: obtaining higher ligand expression/biosynthesis, increased sensitivity (biosensors), increased immunogenicity (new generation vaccines composed exclusively of epitopes). Some of these methods are based on the construction of plasmids carrying multiple joined genes. Strategies enabling an ordered, head-to-tail arrangement of monomeric peptides are preferred over head-to-head or random arrangement. Head-to tail arrangement of cloned multimers provides stabilization of the recombinant DNA plasmid containing concatemeric polymeric DNA. Previous methods (used thus far for the construction of concatemeric, coding DNA) suffered from either the inability or great difficulty in joining DNA segments, while at the same time maintaining continuity of the formed Open Reading Frame. This prevented the formation of the final desired product – concatemeric polypeptides, containing multiple bioactive monomers of the same amino acid sequences with a pre-programmed function, without off-frame segments. This paper presents a novel genetic engineering method for the construction of concatemeric DNA, RNA and finally a protein, containing up to 500 copies of bioactive peptides, which are joined in perfect fusion for in-frame translation of the artificial protein (Fig. 1). This system is based on: specific amplification-expression DNA vectors, containing a universal DNA fragment amplification module, a Type IIS REase, SapI, and T4 DNA ligase [2], [3], [4], [5]. The presented method has numerous potential medical and scientific applications and can also be used in bioprocessing. The technology described is or has been protected by: originally Polish, PCT, EU, USA, Indian, Japanese, Israeli, Chinese patent applications (2014–15), followed by granted Polish patent no. 228341, (2018) [[4], [5]] EU patent (aplication no. EP 15738474.4), (2020) , USA (application no. US2017/0095553 A1), (2020), Indian (application no. 201647039411) (2020)and Japanese (application no. 2017-507091) (2020).

## Materials

### Bacterial strains

Used for plasmid DNA cloning and purification:

*Escherichia coli (E. coli)* TOP10 [F^−^ *mcrA* Δ(*mrr*-*hsdRMS*-*mcrBC*) Φ80*lacZ*Δ*M15* Δ*lacX74 recA1 araD139* Δ(*ara-leu*)*7697 galU galK rpsL* (Str^R^) *endA1 nupG*], Life Technologies (Gaithersburg, MD, USA)
*E. coli* Endura™ [*recA13 supE44 ara-14 galK2 lacY1 proA2 rpsL20* (Str^R^)*xyl-5 λ^–^ leu mtl-1* F*^–^ mcrB mrr hsdS20(r_B_^–^, m_B_^–^)*], Lucigen Corp. (Middleton, USA)
*E. coli* DH5alfa MCR [F^−^ φ*80lacZ*∆*M15* ∆(*lacZYA*-*argF*) *U169 deoR recA1 endA1 hsdR17* (r_K_^−^, m_K_^+^) *phoA supE44* λ^-^ *thi-1 gyrA96 relA1 Mrr*^−^], New England Biolabs (Ipswich, MA, USA)
*E. coli* K2 JM109 [F´ *traD36 proA*^+^*B*^+^ *lacI*^q^ Δ(*lacZ*)*M15*/ Δ(*lac*-*proAB*) *glnV44 e14*^-^ *gyrA96 recA1 relA1 endA1 thi hsdR17*], New England Biolabs (Ipswich, MA, USA)

*Used for gene expression and protein biosynthesis*

*E. coli* Invitrogen™ BL21(DE3), [F^–^ *ompT hsdS_B_ (r_B_^−^, m_B_^−^) gal dcm* λ(DE3)]
*E. coli* Invitrogen™ BL21 Star(DE3) [F^–^ *ompT hsdS_B_ (r_B_^−^, m_B_^−^) gal dcm rne131* λ(DE3)]
*E. coli* Agilent Technologies BL21-Gold(DE3) [F^–^ *ompT hsdS (r_B_^−^, m_B_^−^) dcm*^+^ Tet^R^ *gal* λ(DE3) *endA* Hte]

The strains were from Thermo Fisher Scientific (Waltham, MA, USA).

### Media and reagents

REases SapI, BspQI, SmaI were from New England Biolabs (Ipswich, MA, USA). Marathon DNA polymerase was from A&A Biotechnology (Gdynia, Poland). LguI, T4 DNA Ligase, T4 DNA Polymerase, Shrimp Alkaline Phosphatase (SAP), 100 bp and 1 kb DNA and protein ladders were from Thermo Fisher Scientific Baltics UAB (Vilnus, Lithuania) and GE Healthcare (Uppsala, Sweden).

Bacterial media components were from BTL (Lodz, Poland). Agarose was from Bioshop (Burlington, Canada). DNA purification kits and high resolution agarose (cat. AG42-005) were from Blirt (Gdansk, Poland) and Thermo Fisher Scientific Baltics UAB (Vilnus, Lithuania). Minute™ Protein/Nucleic Extraction Kit was from Invent Biotechnologies, Inc. (Plymouth, USA). The oligos chemical synthesis was performed at Genomed (Warsaw, Poland) or Sigma Aldrich (St. Louis Missouri, MO, USA). The gene synthesis was performed at Bio Basic (Markham ON, Canada) and Genescript (Piscataway NJ, USA). Other reagents were from Avantor Performance Materials Poland S.A. (Gliwice, Poland), AppliChem Inc. (St. Louis Missouri, MO, USA) or Fluka Chemie GmbH (Buchs, Switzerland).

## Software

The genetic maps of the DNA vectors and recombinant constructs were prepared using SnapGene software version 4.1 (http://www.snapgene.com).

### DNA amplification-expression pAMP vectors for temperature-regulated concatemeric protein biosynthesis in *E. coli* cytoplasm

The pAMP vectors were designed on the basis of the *p15A* origin vector pACYC184 [1] and its derivative pRZ4737 (W. S. Reznikoff) [2,3] (GenBank: MK606505, MK606506, MK606507, MK606519, MK606520, MK651654). All pAMP DNA vectors contain: (*i*) a strong, temperature-regulated bacteriophage lambda *pR* transcription promoter, (*ii*) a bacteriophage lambda *cI857ts* repressor gene for host-independence of the vector, (*iii*) a DNA fragment amplification module, with two convergent SapI sites, separated with a SmaI site, for ordered, in-frame, head-to-tail amplification of DNA fragments, resulting in the assembly of an artificial, continuous, multimeric ORF and (*iv*) chloramphenicol resistance gene. The amplifying modules from pAMP vectors are presented inFig. 2. DNA sequences of the pAMP vectors are provided in Supplemental data.

**Fig. 2:**
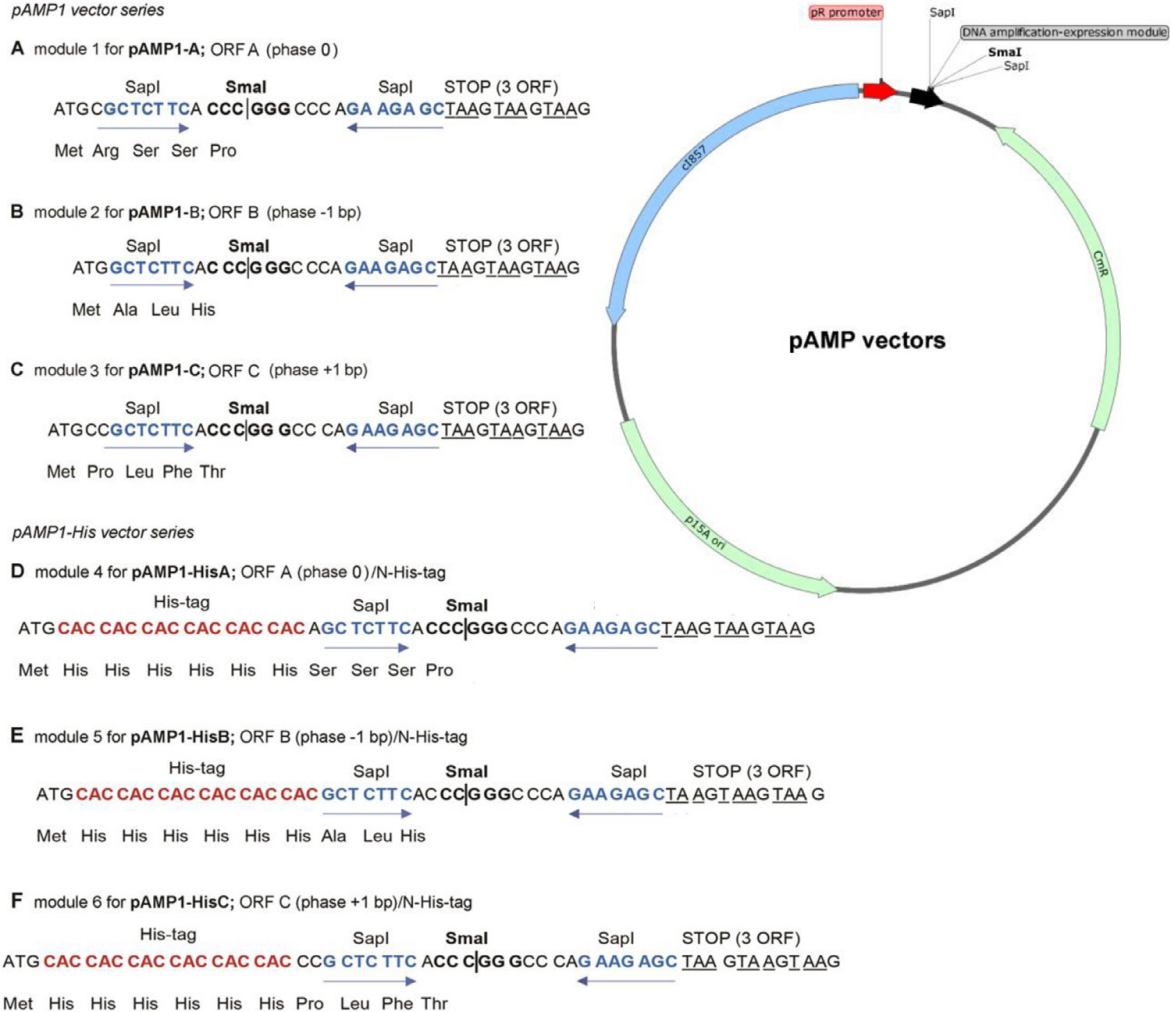
A series of pAMP DNA vectors. The amplification modules differ in their possibility to manipulate three reading frames, as well as the presence or absence of a His6 tag. Optionally, the modules also allow for cloning into the SmaI site, although SapI is preferred. DNA sequences of the vectors have been deposited in the GeneBank database: pAMP1-A (MK651654), pAMP1-B (MK606505), pAMP1-C (MK606506), pAMP1-HisA (MK606507), pMAP1- HisB (MK606519), pAMP1-His C (MK606520).

### DNA amplification-expression pET21AMP-HisA vector for IPTG-regulated concatemeric protein biosynthesis in *E. coli* cytoplasm

**Fig. 3:**
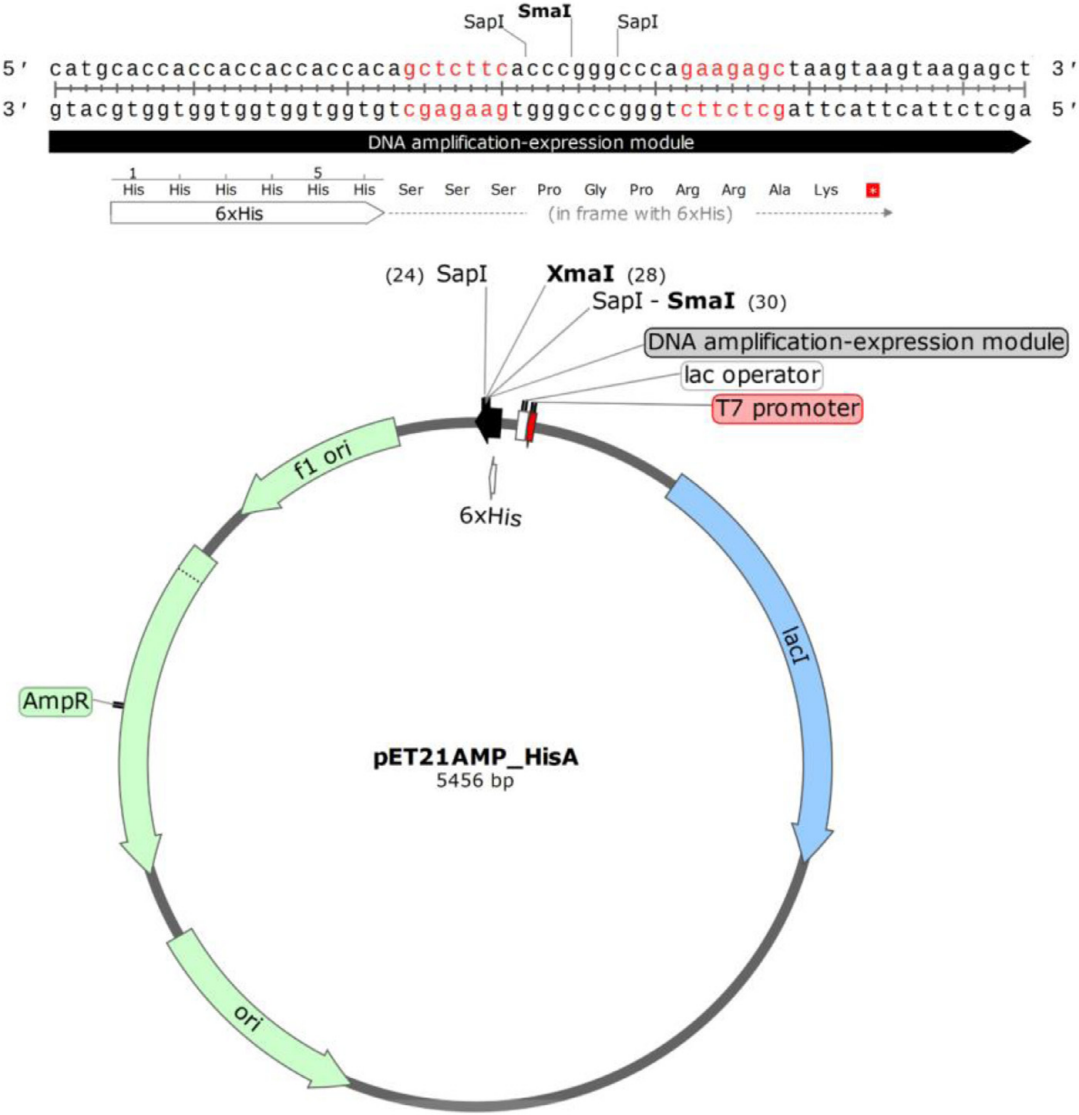
Scheme of pET21AMP_HisA DNA vector and its amplification-expression module.

The pET21AMP-HisA vector was constructed using the pET-21d(+) expression vector (Novagen, EMD Millipore Corporation) as a template for plasmid modification. The pET21AMP-HisA DNA vector (GenBank MK606521) contains: (*i*) a strong, IPTG-regulated T7-lac transcription promoter, (*ii*) *colE1 ori*, (*iii*) a DNA fragment amplification module HisA, with two convergent SapI sites for in-frame, head-to-tail amplification of DNA fragments, resulting in assembly of an artificial, continuous, multimeric ORF and (*iv*) ampicillin resistance gene (Fig. 3). DNA sequence of the pET21AMP-HisA DNA vector is provided in Supplemental data.

### DNA amplification-expression pET28AMP_SapI-Ubq vectors for IPTG-regulated cytoplasmic biosynthesis of concatemeric fusion proteins with an N-terminal ubiquitin

**Fig. 4:**
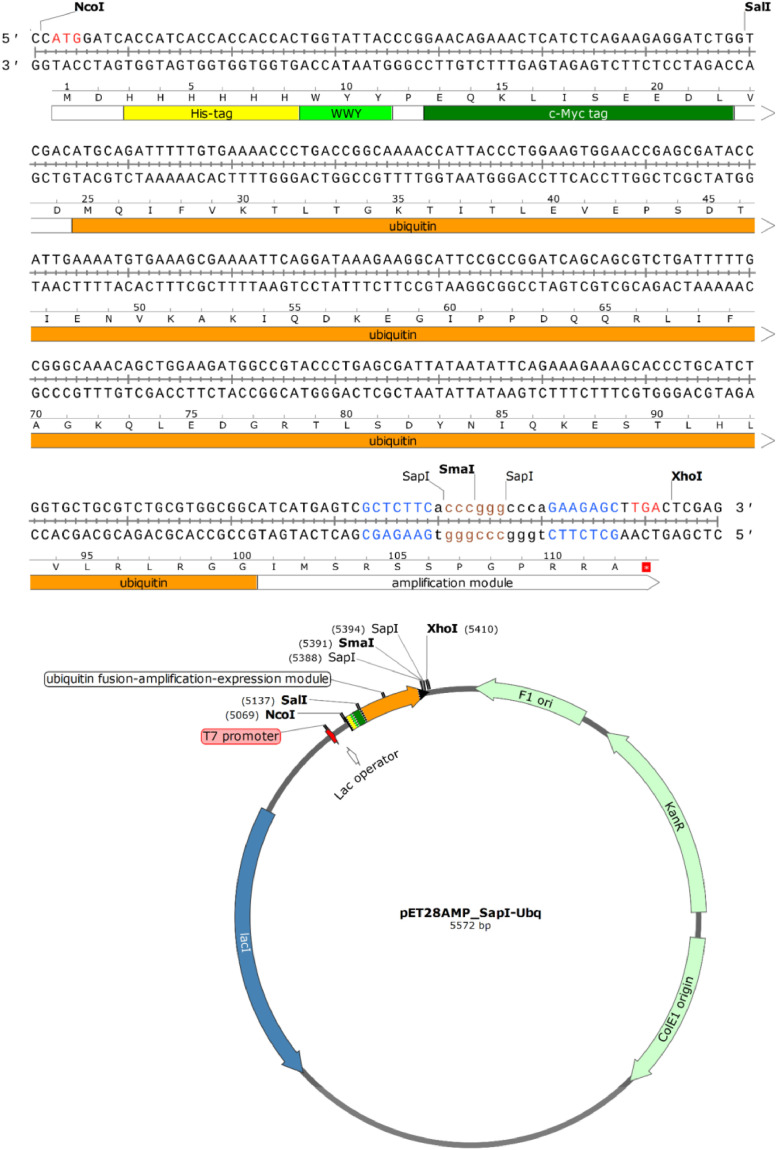
Scheme of pET28AMP_SapI-Ubq DNA vector and its amplification-expression module.

The pET28AMP_SapI-Ubq vector was constructed using the pET-28d(+) expression vector (Novagen, EMD Millipore Corporation) as a template for plasmid modification [2,3]. The SapI and SmaI recognition sequences (originally present in pET-28d(+) vector: positions 3108 and 4300, respectively) were eliminated by site-specific DNA mutagenesis [2,3]. The pET28AMP_SapI-Ubq DNA vector (GenBank MK606527) contains: (*i*) a strong, IPTG-regulated T7-lac transcription promoter, (*ii*) *colE1 ori*, (iii) a DNA fragment amplification module His6_c-Myc_WYY_ubiquitin_SapI-Sma-SapI, enabling ubiquitin gene fusion and (iv) kanamycin resistance gene (Fig. 4). DNA sequence of pET28AMP_SapI-Ubq vector is provided in Supplemental data. The ubiquitin domain can be easily removed from a fusion protein by deubiquitinating proteases [6].

### Amplification-expression-secretion pET28AMP_PhoA and pET28AMP_MalE vectors for IPTG-regulated biosynthesis of concatemeric proteins exported to the *E. coli* periplasm

**Fig. 5:**
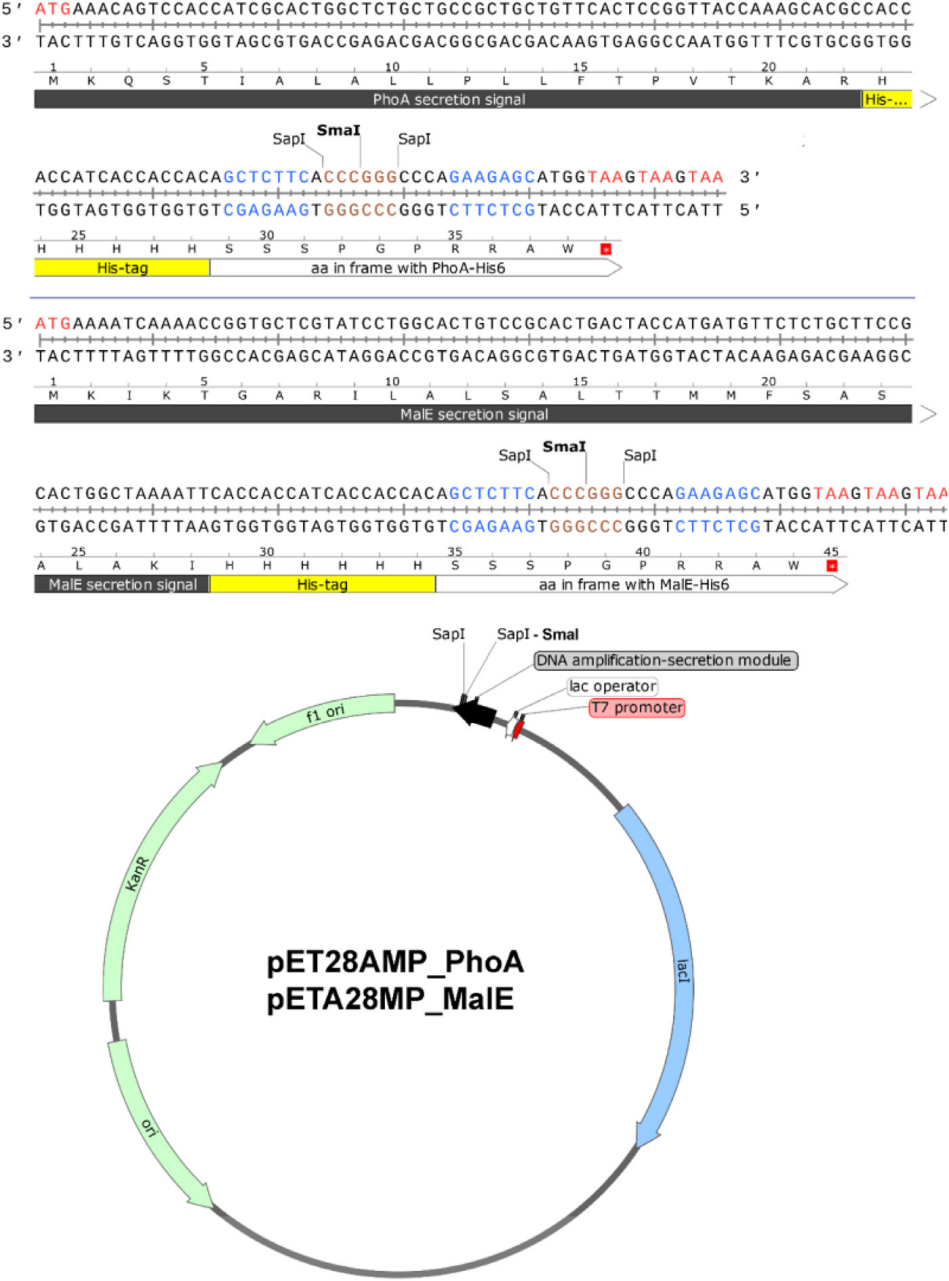
Scheme of pET28AMP_PhoA and pET28AMP_MalE DNA vectors and their amplification-secretion modules.

The pET28AMP_PhoA and pET28AMP_MalE vectors were constructed using the pET-28d(+) expression vector (Novagen, EMD Millipore Corporation) as a template for plasmid modification [2,3]. The SapI and SmaI recognition sequences (originally present in pET-28d(+) vector: positions 3108 and 4300, respectively) were eliminated by site-specific DNA mutagenesis [2,3]. The pET28AMP_PhoA DNA vector (GenBank MK606526) contains: (*i*) a strong, IPTG-regulated T7-lac transcription promoter, (*ii*) *colE1 ori*, (*iii*) a DNA fragment amplification-secretion module His6_PhoA_SapI-Sma-SapI, and (iv) kanamycin resistance gene (Fig. 5). The pET28AMP_MalE vector (GenBank MK606522) is an equivalent to the pET28AMP_PhoA DNA vector, except that it contains an alternative amplification-secretion module: His6_MalE_SapI-Sma-SapI (Fig. 5). DNA sequences of the vectors are provided in Supplemental data.

## Method details

The rapidly developing field of synthetic biology is generating insatiable demands for synthetic genes. Depending on their application, newly designed genes may contain repetitive DNA fragments, which significantly impair their chemical synthesis. The capability of generating DNA molecules of any sequence or size is important especially for biomedical research. Here we present a significant improvement from an earlier strategy [7] that enables the formation of artificial, continuous, multimeric ORFs, concatemeric proteins of desired length and a monomer copy number using four series of specialized amplification-expression DNA vectors, equipped with a universal DNA amplification module. The module contains two convergent DNA recognition sequences of the Type IIS REase SapI, separated with a SmaI site for the insertion of any DNA fragment. It may be easily modified and introduced, as desired, into various DNA vectors, containing alternative origins of replication, antibiotic resistance genes, transcriptional promoters and translation initiation signals.

The presented method has numerous potential applications, especially in the pharmaceutical industry and tissue engineering, including vaccines and drug delivery systems production, as well as mass-production of peptide-derived biomaterials. The technology enables easy and efficient construction of artificial, concatemeric genes, greatly exceeding current chemical gene synthesis capabilities.

### General scheme for directional DNA fragment amplification and concatemers construction

**Fig. 6:**
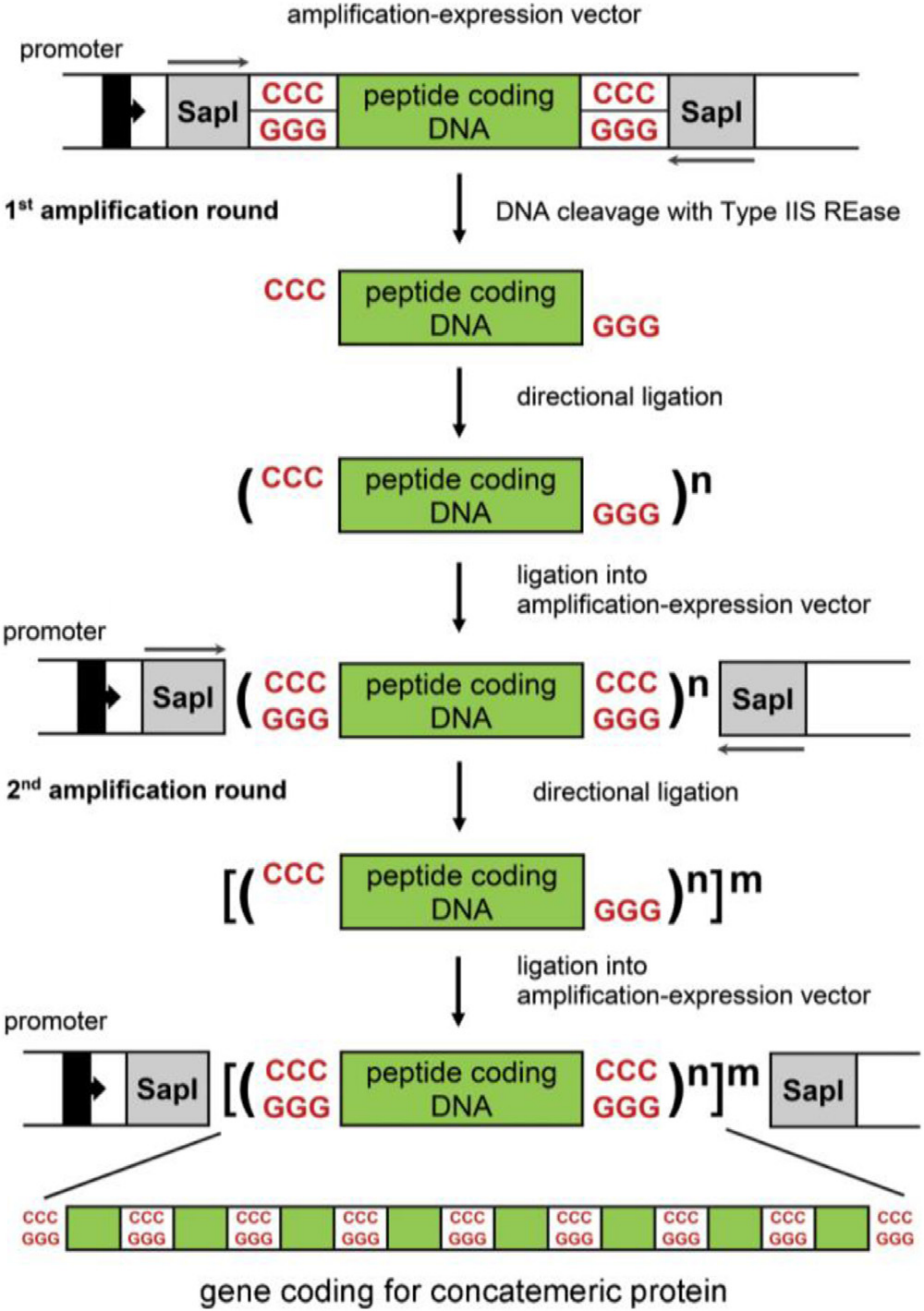
Diagram showing the principle of the developed DNA fragment amplification-expression method.

Four types of DNA vectors were designed for the needs of the DNA fragment amplification methodology: *i)* pAMP series and pET21AMP DNA vector for concatemeric protein biosynthesis in *E. coli* cytoplasm, *ii*) pET28AMP_SapI-Ubq vector for cytoplasmic biosynthesis of concatemeric fusion proteins with an N-terminal ubiquitin and *iii*) pET28AMP_PhoA or pET28AMP_MalE vectors for biosynthesis of concatemeric proteins secreted to the *E. coli* periplasm. One should follow the same general protocol for all the designed DNA vectors. Protocol stages are as follows: (*i*) design or selection of the DNA fragment (monomer) to be amplified; (*ii*) chemical synthesis of DNA, PCR amplification or excision of the monomer using restriction endonucleases; (*iii*) addition of asymmetric, preferably 5′-CCC-3′/5′-GGG3’, SapI 3-nt cohesive ends at 5′ and 3′ termini of the monomer. This can be achieved by introducing SapI recognition sequences during chemical synthesis of the monomer, PCR amplification or with the use of the vector's built-in SapI sites, after cloning the restriction fragment; (*iv*) purification of the DNA monomer equipped with SapI 3-nt cohesive ends; (*v*) directional self-ligation of DNA monomers in directional, head-to-tail orientation; (*vi*) ligation of a mixture of the formed concatemers or a selected gel-purified concatemer back into a SapI-cleaved amplification vector; (*vii*) selection of bacterial clones containing a concatemer with the desired number of monomers; (*viii*) direct biosynthesis of the protein encoded by the obtained DNA concatemer using the vector's strong promoters or excision of the concatemer, equipped with SapI-cohesive ends and repetition of steps (*iv*)-(*viii*), until a desired number of monomer copies within a concatemer is obtained (Fig. 6).

### Experimental procedure for directional head-to-tail amplification of a short DNA fragment

1. Design a synthetic dsDNA fragment encoding a bioactive peptide to be concatemerized.

***Note 1:*** *A synthetic DNA fragment should be equipped with two convergent SapI recognition sequences for straightforward, in-frame, head-to-tail amplification of the designed DNA fragment, resulting in the assembly of an artificial ORF. SapI cleavage generates 3-nt, single-stranded 5′ cohesive ends 5′-CCC-3′/5′-GGG-3′.*

***Note 2:*** *The designed dsDNA should have at least three base pairs from the end followed by SapI recognition sequences. We recommend adding GC clamps: the presence of G or C bases within the last three bases from the 3′ end of the oligonucleotides (GC clamp) helps promote specific binding at the 3′ end and improves SapI cleavage. Moreover, a GC clamp aids in specificity of the priming and therefore contributes to the overall efficiency of the PCR reaction.*

2. Optimize a peptide-coding DNA sequence for efficient gene expression in *E. coli*.
3. Chemically synthesize the designed DNA fragment *de novo*.
4. Prepare a suitable amount of the DNA monomer for concatemerization using:

(a) PCR with properly designed (e.g. containing ssDNA overhangs with SapI sites) forward and reverse primers, followed by SapI cleavage and DNA purification.
(b) SapI excision of the cloned DNA monomer from the recombinant plasmid DNA and purification of the desired restriction fragment.

***Note 3:*** *For option b) one can clone a synthetic DNA fragment into the selected amplification-expression vector using SapI restriction sites. Alternatively, a 5′-phosphorylated dsDNA fragment can be blunt end cloned into a SapI site or SmaI site, filled-in with dCTP/dGTP, of the selected vector's amplification-expression module. The obtained recombinant DNA constructs can be used to transform E. coli. A correct orientation of the cloned insert should be verified by DNA sequencing. The recombinant plasmid DNAs can be purified from the obtained bacterial clones in the desired amount and used for further SapI cleavage. SapI restriction fragments can be separated electrophoretically and the desired DNA fragment should be isolated from the gel, preferably using electroelution. For further directional amplification, we recommend purification of the isolated DNA fragment using phenol:chloroform extraction and ethanol precipitation. In our case this strategy was more effective than directional ligation of the PCR-amplified and SapI-cleaved DNA fragments.*

***Note 4:*** *Perform SapI DNA cleavage for 2* *h at 37* °*C, in a suitable reaction volume. Composition of the reaction mixture: 20* *mM Tris-acetate (*pH *7.9 at 25* °*C), 10* *mM magnesium acetate, 50* *mM potassium acetate, 100* *µg/ml BSA, 20* *µg of the DNA fragment, 5 units of SapI. Inactivate the enzyme by 20* min *incubation at 65* °*C. Separate the resulting SapI restriction fragments using polyacrylamide gel electrophoresis (PAGE) in 10% polyacrylamide gel in TBE buffer. Cut out the suitable SapI restriction fragment from the gel and purify it using electroelution or a commercially available DNA purification kit (for example: Minute™ Protein/Nucleic Extraction Kit (Invent Biotechnologies, Inc.)*

***Note 5:*** *One can use other commercially available SapI isoschizomers: BspQI or LguI following the manufacturer's instructions.*

5. Perform the first round of directional ligation.

(a) Subject the purified, SapI-cleaved DNA fragment (1.5 µg) to autoligation in vitro at 16 °C using 0.01 Weiss units of T4 DNA ligase with a reaction volume of 72 µl. Reaction buffer: 40 mM Tris–HCl, 10 mM MgCl_2_, 10 mM DTT, 0.5 mM ATP (pH 7.8 at 25 °C). Take samples at reaction time intervals of 5, 10, 20, 40, 80 and 160 min.
(b) Analyse ligation products by polyacrylamide or agarose electrophoresis. A series of DNA segments of increasing length, forming directional concatemers of the polymerised gene, should be obtained.
(c) Purify concatemer(s) of desired length from the gel.
(d) Clone the concatemer or concatemers mixture into the selected, SapI-linearized and dephosphorylated DNA vector, preferably with thermolabile Shrimp Alkaline Phosphatase (SAP).

***Note 6:*** *For DNA cloning, the following reaction composition and conditions can be used: 40* *mM Tris–HCl (*pH *7.8 at 25* °*C), 10* *mM* MgCl_2_*, 10* *mM DTT, 0.5* *mM ATP, 540* *ng of the autoligated insert DNA mixture, 270* *ng of the SapI-linearized and dephosphorylated vector DNA, 1 Weiss unit of T4 DNA Ligase. Perform the reaction at 16* °*C for 16* *h in a 90* *µl reaction volume. Purify the resulting ligation products and transform them into electrocompetent E. coli cells. One can test various E. coli strains. However, we recommend E. coli strains well tolerating DNA repeats, such as DH5alfa, Top10, JM109 and Endura™. We recommend testing several different E. coli strains to get the maximum possible monomer copy number within a concatemer. Various E. coli strains may tolerate different lengths of concatemers, depending on the type and sequence of the designed construct.*

6. Subject the obtained recombinant constructs directly to expression of the artificial gene, coding for a concatemeric protein or alternatively perform another amplification cycle to obtain longer concatemeric genes.

***Note 7: Alternative amplification.*** In order to further boost DNA synthesis capability, one can design and try to chemically synthesize an artificial gene, encoding from several to several dozen copies of the selected peptide (possibilities for chemical DNA synthesis of a given repetitive DNA sequence are the only limit in this case). Such a synthetic, repetitive gene can be cloned into the selected, SapI-linearized and dephosphorylated amplification-expression vector and used as a ‘monomer’ for further amplification as described in *Note 3*.

### Experimental procedure for directional head-to-tail amplification of a long DNA fragment

For directional, head-to-tail ligation of longer DNA fragments a slight modification concerning addition of PEG4000 to the ligation buffer is required to decrease circularization of the obtained ligation products.

1. In the first stage, prepare a suitable DNA ‘monomer’, equipped with 3-nt, single-stranded 5′ cohesive ends CCC/GGG by SapI cleavage and gel-purification, as described previously. Then, perform autoligation of the prepared DNA ‘monomers’ for 16 h at 16 °C, in a reaction volume of 20 µl. The following reaction composition can be used: 2 µg of the excised DNA fragment (‘monomer’), 40 mM Tris–HCl (pH 7.8 at 25 °C), 150 mM NaCl, **20% PEG4000**, 10 mM MgCl_2_, 10 mM DTT, 0.5 mM ATP, 0.01 or 0.1 Weiss units of T4 DNA ligase. Samples should be taken at reaction time intervals of 15, 30, 45, 60 min. All the reaction products should be combined and purified.
2. In the second stage, clone the obtained DNA mixture into a selected, SapI-linearized and dephosphorylated amplification-expression DNA vector. For this purpose, the ligation reaction can be performed in a volume of 20 μl containing: 40 mM Tris–HCl (pH 7.8 at 25 °C), 10 mM MgCl_2_, 10 mM DTT, 0.5 mM ATP, 400 ng of the autoligated insert DNA mixture, 50 ng of the SapI-linearized and dephosphorylated pAMP1-HisA vector DNA,1 Weiss unit of T4 DNA Ligase. The ligation reaction can be conducted at 22 °C for 1 h. Then, the T4 DNA ligase should be inactivated by incubation at 70 °C for 5 min. The resulting DNA ligation products should be purified, ethanol precipitated and used for *E. coli* transformation.

## Method validation

A validation of the presented method is described by Skowron et al. [2] and the corresponding Data in Brief article [3]. The technology has been validated so far by amplification of four DNA fragments, encoding the following peptides: (i) TKPTDGNGP (MSEC_2019_1496), (ii) TSRGDHELLGGGAAPVGG (MSEC_2019_1496; patent application P.427146), (iii) RGD and RGDGG (patent application P.425131) and (iv) RLIDRTNANFLGGGAAPVGGG (patent application P.427146). We managed to obtain up to 500 copies of our test model (the TKPTDGNGP peptide), using two rounds of amplification [2]. One should note, however, that the maximum obtainable monomer copy number within a concatemer may be lower than in the case of the mentioned test model [2] and strongly depends on the DNA sequence and the size of the DNA fragment to be concatemerized. Moreover, one should realize that some of the constructed concatemeric genes may not be efficiently expressed in *E. coli*. This may require a development of new strategies: improving concatemeric gene transcription and its mRNA stability, reducing toxicity or increasing solubility of the resulting proteins, as well as using alternative prokaryotic or eukaryotic expression systems. Examples of such strategies are: (*i*) N-terminal fusion with ubiquitin described herein or (*ii*) secretion of concatemeric proteins into *E. coli* periplasm [2]. It is worth mentioning that the DNA amplification-expression modules presented herein (Fig. 2-5) can be easily transferred to other DNA vectors, if necessary.

## Conclusions

1. A protocol for a new DNA fragment amplification-expression technology, devised for the production of artificial genes, encoding concatemeric RNAs and proteins with a pre-programmed nucleotide and aa sequence, is provided. The technology directs formation of ordered polymers or co-polymers, containing 500 or more copies of repeated monomeric units of DNA, RNA or peptides within a concatemer.
2. The ordered polymerisation of a DNA fragment, encoding a peptide with a pre-programmed biological or chemical function, can improve the desirable function of the resulting artificial polypeptide/protein. Such concatemeric proteins may serve for a number of constructions, exemplified by: (*i*) enhanced antigens - a new generation of vaccines; (*ii*) concatemeric proteins containing modules binding rare and/or toxic metal ions for their industrial recovery, environment remediation or the removal of toxins from the body; (*iii*) novel reservoirs for enzyme cofactors that can modulate particular enzymatic activity; (*iv*) reservoirs for peptide hormones; (*v*) protective, therapeutic concatemeric proteins, containing peptide activators or inhibitors for tissue regeneration or treatment of molecular, microbial and viral diseases; (*vi*) reservoirs for polymerised micro RNA, antisense nucleic acids against genetic, molecular, microbial and viral diseases.

## Declaration of Competing Interest

The authors declare that there is no conflict of interest regarding the publication of this article.

## Appendix Supplementary materials

Image, application 1
